# Virtual and augmented reality in intensive care medicine: a systematic review

**DOI:** 10.1186/s13613-023-01176-z

**Published:** 2023-09-11

**Authors:** Dominika Kanschik, Raphael Romano Bruno, Georg Wolff, Malte Kelm, Christian Jung

**Affiliations:** 1 Department of Cardiology, Pulmonology, and Vascular Medicine, Medical Faculty, Heinrich-Heine-University, University Hospital Duesseldorf, Duesseldorf, Germany; 2 Cardiovascular Research Institute Duesseldorf (CARID), Medical Faculty, Heinrich-Heine University, Duesseldorf, Duesseldorf, Germany

**Keywords:** Augmented reality, Virtual reality, Mixed reality, Intensive care medicine, Imaging

## Abstract

**Background:**

Virtual reality (VR) and augmented reality (AR) are rapidly developing technologies that offer a wide range of applications and enable users to experience digitally rendered content in both physical and virtual space. Although the number of studies about the different use of VR and AR increases year by year, a systematic overview of the applications of these innovative technologies in intensive care medicine is lacking. The aim of this systematic review was to provide a detailed summary of how VR and AR are currently being used in various areas of intensive care medicine.

**Methods:**

We systematically searched PubMed until 1st March 2023 to identify the currently existing evidence for different applications of VR and AR for both health care providers in the intensive care unit and children or adults, who were in an intensive care unit because of a critical illness.

**Results:**

After screening the literature, a total of 59 studies were included. Of note, a substantial number of publications consists of case reports, study plans or are lacking a control group. Furthermore, study designs are seldom comparable. However, there have been a variety of use cases for VR and AR that researchers have explored. They can help intensive care unit (ICU) personnel train, plan, and perform difficult procedures such as cardiopulmonary resuscitation, vascular punctures, endotracheal intubation or percutaneous dilatational tracheostomy. Patients might benefit from VR during invasive interventions and ICU stay by alleviating stress or pain. Furthermore, it enables contact with relatives and can also assist patients in their rehabilitation programs.

**Conclusion:**

Both, VR and AR, offer multiple possibilities to improve current care, both from the perspective of the healthcare professional and the patient. It can be assumed that VR and AR will develop further and their application in health care will increase.

**Graphic Abstract:**

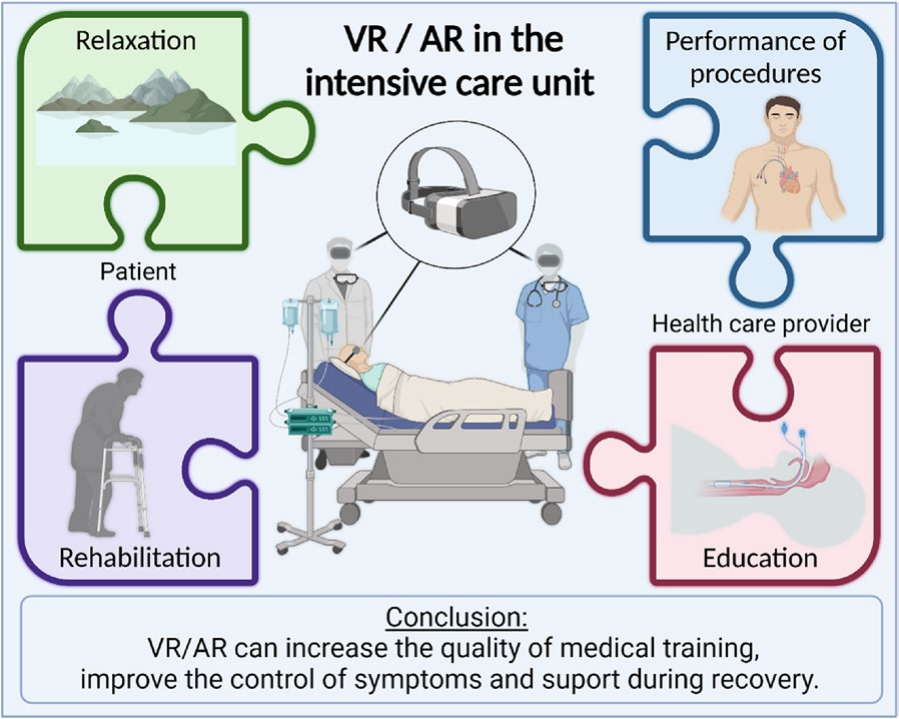

## Background

Virtual reality (VR) and augmented reality (AR) are emerging technologies that allow various applications, ranging from immersive entertainment or educational experiences to medical care. VR is defined as the user’s complete immersion into a virtual three-dimensional environment, while AR retains the connection to the real world but supplements it with virtual elements to increase information [1]. Both VR and AR necessitate special VR/AR glasses for the user. Medical applications are growing and there are already areas that have been intensively researched, such as cardiovascular care [2] or neurosurgery [3]. The technologies are also increasingly being used in intensive care medicine and might positively influence this area of medicine from the perspectives of both medical staff and patients [4]. In a safe environment, VR can help health care providers in acquiring and practice complex intensive care procedures [5]. Augmented reality can also support the user both before and during procedures by integrating various additional information into reality [6]. From the patient´s point of view, VR can help to reduce stress during the stay in the intensive care unit through different means, such as distraction from pain, for both adults [7] and children [8]. In addition, by combining virtual reality and gaming, it is possible to improve cognitive and motor skills [9]. Thus, VR and AR could potentially be used at different time points by several users and for different purposes.

The present systematic review presents the current status of the application of VR and AR in critical care medicine. Based on a literature review, we summarized the current state-of-the-art.

## Methods

### Literature search

We systematically searched PubMed databases for publications up until 1st March 2023, applying the following keywords: “VR” and “ICU”, “virtual reality” and “ICU”, "virtual reality" and "critical care", “virtual reality” and “intensive care unit”, "augmented reality" and "ICU, “augmented reality" and "critical care", "augmented reality" and "intensive care", “mixed reality” and “ICU”, "mixed reality" and "critical care", “mixed reality” and “intensive care unit” (Appendix 1) to identify all published studies reporting on the application of virtual or augmented reality in the intensive care unit.

### Eligibility and selection criteria

Eligible articles were: randomized controlled trials, nonrandomized trials, observational studies (cases and controls, cohort, and cross-sectional studies), proof-of-concept studies, study protocols, and case reports or series. All studies that met the following criteria were included: (1) type of participants: subjects were either health care providers in the intensive care unit or children or adults, who were in an intensive care unit because of a critical illness. (2) Type of interventions: VR or AR (3) Language: studies published in English or German, both in full text or abstract-only formats.

### Data abstraction

Three independent reviewers screened all articles using the above-mentioned inclusion criteria. An independent fourth investigator was involved in the case of discrepancies in the extraction and assessment of the data. The following data were abstracted: author’s name, year of publication, study type, sample size, inclusion criteria, patient characteristics (age, medical background, and treatment), use of AR/VR, frequency of application, and outcomes.

### Data synthesis

The key characteristics and results of included studies were summarized and synthesized using tables and complemented by a qualitative summary. This study was conducted and reported following the PRISMA (Preferred Reporting Items for Systematic Reviews and Meta-Analyses) guidelines for reporting systematic reviews [10].

## Results

**Fig. 1:**
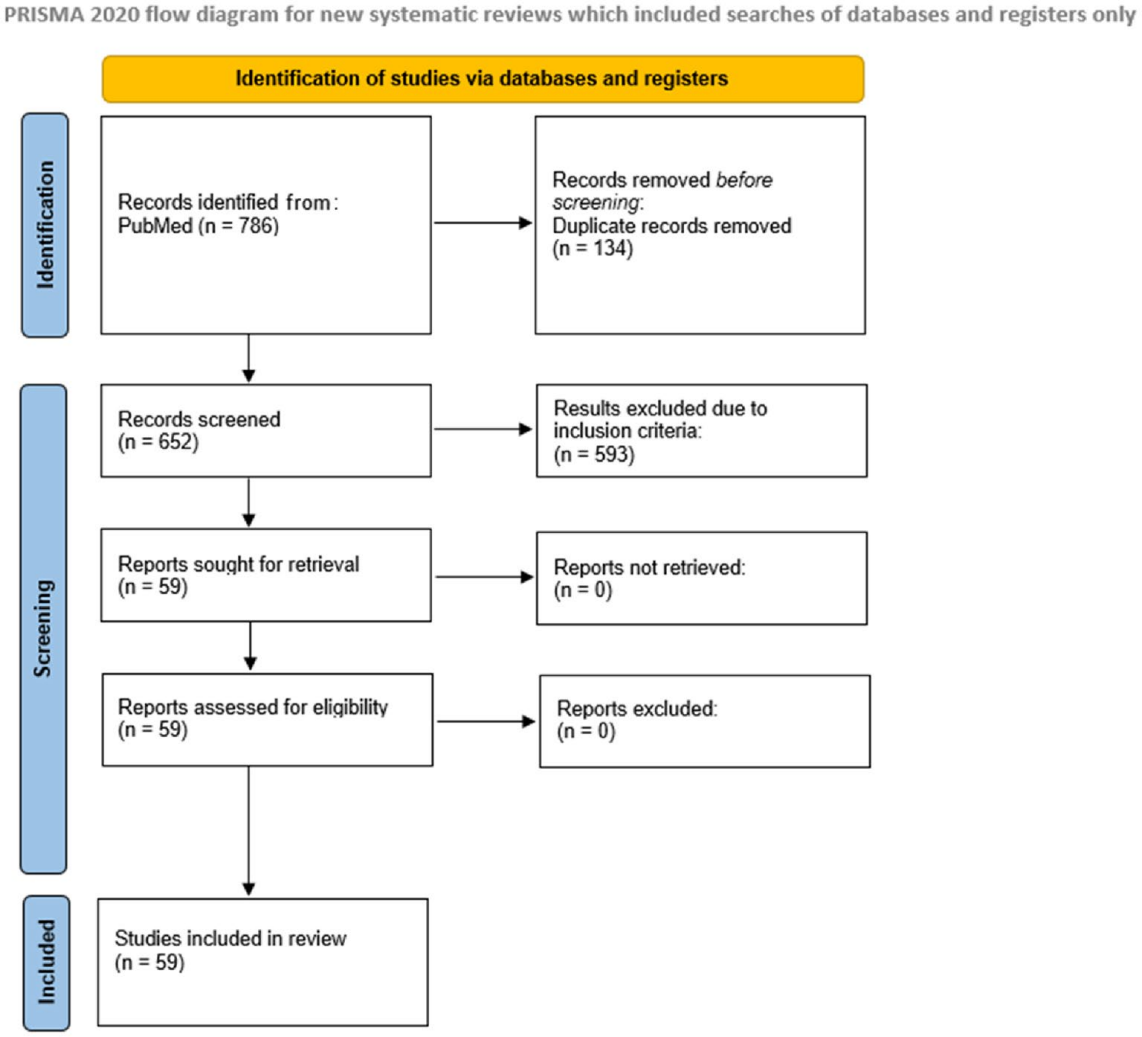
Study flowchart

The initial search strategy identified 786 articles (Fig. 1). After the screening on predefined criteria and removal of duplicates, 59 studies were included.

There were 48 studies about the application of VR: 12 randomized control trials, 2 nonrandomized trials, 26 observational studies, 2 proof-of-concept studies, 4 study protocols and 2 case reports, and 11 studies about the use of AR: 3 randomized control trial, 7 observational studies, and 1 case series.

Table 1 and Table 2 summarize all studies about the use of VR (Table 1) and AR (Table 2) in intensive care medicine that were included in the search strategy. So all relevant studies are described and commented on in a systematic review. Part 1 focuses on VR and Part 2 on AR. For each perspective, we discuss different applications, both in the adult and pediatric intensive care unit.

**Table 1:** Systematic review of virtual reality in intensive care medicine

**Authors (year, country)**	Sample size (intervention/ control)	Study type	Quality of evidence[75]	VR user	Age (mean ± SD) / median (Range)	VR dosage (frequency)	The timing of applying VR	Experimental group intervention	Control group intervention	Results
Adult intensive care medicine										
Laghlam et al. (2021, France) * [25]	200 (99/101)	Randomized, prospective trial	2	ICU patients	68.0 (60.0–74.8)	Surgery time	During the removal of chest drains after cardiac surgery	VR session	Inhaled equimolar mixture of N_2_O and O_2_ (Kalinox®)	VR did not reach the statistical requirements for a proven non-inferiority vs. Kalinox® in managing pain and anxiety during chest drain removal (mean difference in analgesia/nociception index − 0.6 [− 3.6 to 2.4], including the non-inferiority margin of 3). Moreover, VR was less effective based on Numeric Rating Scale (VR: 5.0 [3.0–7.0] vs. Kalinox: 3.0 [2.0–6.0], *p* = 0.009)
Lee and Kang (2020, South Korea) * [31]	48 (24/24)	Randomized controlled trial	2	ICU patients	66	30 min (once)	30 min before bedtime (9–11 PM) on the day of ICU admission	Meditation using a head-mounted display for virtual reality	No meditation	The experimental group reported significantly higher subjective sleep quality than the control group (*p* = 0,002). Activity tracker assessment indicated that total sleep time and light sleep time did not differ between the groups (*p* = 0,071). However, the awake time was shorter, deep sleep time was longer and sleep efficiency (*p* = 0.008) was significantly higher (*p* = 0.018) in the experimental group than in the control group
Vlake et al. (2021, Netherlands) * [50]	104 (57/47)	Randomized controlled trial	2	ICU patients	61	10 min	Shortly after being discharged from the ICU, while still being treated in the hospital ward	ICU-VR group receiving ICU-VR	Control VR group receiving a nature VR environment	ICU-specific virtual reality patients experienced higher immersion, cybersickness scores were low, and no changes in vital signs were observed. They also reported reduced posttraumatic stress disorder and depression scores and better mental health from 2 days until 1 month after initial exposure(Short Form-12 Mental 2-Component Scale: ICU-specific virtual reality, 57 [36, 67] vs control virtual reality, 47 [26, 63]; *p* < 0.01). Six months after exposure, this effect was still present for post-traumatic stress disorder and depression, but not for mental quality of life
Vlake et al. (2021, Netherlands) [76]	45 (15/15/15)	Randomized controlled trial	2	Healthy participants	61 (45-75)	10:55 min	n/a	Head-mounted display virtual reality group (*n* = 15)	2D group (*n* = 15), and crossover group (*n* = 15)	ICU-specific virtual reality appears safe and more immersive than 2D, implicating that ICU-specific virtual reality is feasible for clinical use. Volunteers in the crossover group experienced a higher total presence (*p* < 0.001) when using head-mounted display virtual reality, expressed as a higher sense of presence (*p* < 0.001), more involvement (*p* < 0.01), and more experienced realism (*p* < 0.001). One should however be aware of simulator sickness-related symptoms
Navarra-Ventura et al. (2021, Spain) * [45]	72 (34/38)	Randomized controlled trial	2	ICU patients	69.1 [35.7–85.9]	15–20 min	ICU stay	VR-based neurocognitive intervention	Standard ICU care	VR-based neurocognitive stimulation may help improve short-term working memory outcomes. Patients in the VR group had better working memory scores (*p* = 0.009, *d* = 0.363) and showed up to 50% less non-specific anxiety (11.8% vs. 21.1%) and depression (5.9% vs. 10.5%)
Vlake et al. (2022, Netherlands) * [51]	89 (45/44)	Randomized controlled trial	2	ICU patients	58 ± 11	14 min	Post-COVID-19 3, 4, and 6 months after hospital discharge	VR-intervention 3, 4, and 6 months after hospital discharge	Standard follow up	ICU-VR improves satisfaction and reduces the prevalence of psychological distress after ICU treatment. ICU-VR did not improve psychological recovery or quality of life
Merliot‑Gailhoustet et al. (2022, France) * [29]	60 (60/0)	Randomized controlled trial	2	ICU patients	62 [51, 69]	15 min per session	ICU stay	4 relaxation sessions (standard relaxation with television/radio, music therapy, and two virtual reality systems with real motion pictures or synthetic motion pictures)	n/a	VR systems were associated with a significant decrease in overall discomfort, stress, anxiety, pain, and in lack of rest. VR relaxation therapy is a promising, safe, and effective solution to improve overall discomfort in ICU patients
Bodet-Contentin et al. (2022, France) * [21]	88 (88/0)	Randomized controlled trial	2	ICU caregivers	n/a	8 min	Break time in the ICU	A half-hour break time including an 8-min-long VR session	Usual break time	VR reduced the fatigue score after the break time significantly and increased the feeling of disconnection from the work environment
Chiang et al. (2021, Taiwan) * [14]	60 (30/30)	Prospective, controlled, 2:1 randomized pre-pre-postudy	2	Healthcare providers	21—30	15 min	Training	VR-based learning on tracheostomy care	Text-based training	VR training increased self-efficacy, including the aspects of familiarity and confidence, and reduced anxiety about tracheostomy-related knowledge and care skills. The benefits persisted until 3 to 4 weeks later
Colt et al. (2001, United States) * [13]	9 (5/4)	Nonrandomized trial	3	Bronchoscopist novices	n/a	n/a	Before and after 4 h of group instruction and 4 h of individual unsupervised practice	VR to practice performing inspection flexible bronchoscopy	4 skilled physicians performing bronchoscopy without previous VR practice	A short, focused course of instruction and unsupervised practice using a virtual bronchoscopy simulator enabled novice trainees to attain a level of manual and technical skill at performing diagnostic bronchoscopic inspection similar to those of colleagues with several years of experience (speed: *p* = 0,33, accuracy: *p* < 0,05)
Gerber et al. (2019, Switzerland) [77]	33 (33/0)	Observational study	3	ICU patients	63 (32-83)	5 min	3 times: ICU admission, during ICU stay, and 3 months after discharge	Video presenting aquatic worlds and landscapes for 5 min	n/a	VR was recalled better (84,4%) than the rest of the ICU stay (30.3%) and well accepted. The decreased respiratory rate during stimulation indicates a relaxing effect of VR (Pre-3ICU session − 1.34 (3.93) breath/min, *p* = 0,039; ICU session -0,56 (1, 79), *p* = 0.048; Follow-up session − 1.88 (4.65), *p* = 0.021)
Mosso-Vázquez et al. (2014, Mexico) * [23]	67 (67/0)	Observational study	3	Patients after cardiac surgery	n/a	30 min	Within 24 h after cardiac surgery	30-min VR simulation designed for pain management	n/a	A heavy positive correlation existed between breathing rate and Likert ratings (*R*^2^ = 0.259), and a moderate correlation was found between mean arterial pressure and Likert ratings (*R*^2^ = 0.087) and heart rate and Likert ratings (*R*^2^ = 0.049), all of which indicated lower pain and stress within patients
Jawed et al. (2021, United States) * [36]	36 (36/0)	Observational study	3	15 patients / 21 health care providers	Patients 60.8 ± 10.9 health care providers 32.5 ± 7.8	15 min	Any time	15-min session showing a relaxing beach scene	n/a	There was a high level of acceptance of VR, reducing anxiety, with minimal side effects
Markus et al. (2009, United States) * [26]	10 (10/0)	Observational study	3	Burn patients	n/a	6 min (2-9 min)	n/a	Measurement of staff resources needed to implement VR	n/a	A mean of 59 staff time minutes (S.D. 18; range 29–85) was required for set-up, instruction, VR therapy, and cleaning. Set-up required the most time, averaging 23 min Instruction, participation, and clean-up means were 6, 13, and 16 min, respectively
Gerber, et al. (2019, Switzerland) [78]	45 (45/0)	Observational study	3	Healthy participants	59 (22–87)	10 min (two times)	n/a	Two times with the head-mounted display (i.e., virtual nature and urban VR stimulation) and once with the gold standard (control condition), a classical ICU TV screen (movie), with each interaction lasting 10 min	n/a	The results showed that the natural environment had the highest positive and restorative effect (none = 0 and high = 1) on the physiological and psychological state of healthy subjects (mean 0.773; SD 0.142), followed by the urban environment (mean 0.65715; SD 0.187) and the ICU TV screen (mean 0.5854; SD 0.136). VR might reduce sensory overload and deprivation and thus prevent neurocognitive late effects
Gomes et al. (2019, Brasil) * [47]	60 (60/0)	Observational study	3	ICU patients	47 ± 17	6 min	During physical therapy sessions	Nintendo Wii™ (Nintendo of America Inc.™, USA) gaming system	n/a	Virtual rehabilitation elicited light to moderate levels of activity in intensive care unit patients
Haley et al. (2022, United States) * [30]	10 (10/0)	Observational study	3	Mechanically ventilated patients	58 (49-66)	5 min	n/a	VR session with a cinematic video of an outdoor green space or blue space with 360° visual range of motion	n/a	No occurrences of the predefined safety events, and no occurrences of cybersickness. The use of a visual analog scale to measure anxiety levels was feasible for this pilot study
Hoffman et al. (2008, United States) * [27]	11 (11/0)	Observational study	3	Burn patients	27 (9-40)	3 min	During burn wound debridement in the hydrotherapy tank	3-min sessions with no VR distraction (i.e., standard premedication only) and 3-min treatment session with VR in randomized order	n/a	Patients reported significantly less pain when distracted with VR [e.g., "worst pain" ratings during wound care dropped from "severe" (7.6) to "moderate" (5.1), p = 0,015]. The 6 patients who reported the strongest illusion of "going inside" the virtual world reported the greatest analgesic effect of VR on worst pain ratings, dropping from severe pain (7.2) in the no VR condition to mild pain (3.7) during VR (*p* < 0,05)
Nijland et al. (2021, Netherlands) * [20]	66 (66/0)	Observational study	3	ICU nurses	42.4 (12.2)	10 min (no limitation on frequency)	During shift	Navigate through high-quality immersive 360-degree videos of calming natural environments	n/a	Mean perceived stress was lowered by 39.9% after the use of VRelax (mean difference = 14.0, SD = 13.3, *p* < 0.005). The mean score on the Perceived Stress Scale-10 was 11.4 (SD = 6.50), and the mean score on the Connor–Davidson Resilience Scale-10 was 29.0 (SD = 5.51). Sixty-two percent of the ICU nurses thought VRelax was helpful to reduce stress
Ong et al. (2020, United States) * [40]	59 (59/0)	Observational study	3	ICU patients	50 ± 18	5-0.20 min (up to seven sessions)	Up to seven sessions, each at least 24 h apart	Guided meditation for breath control and progressive relaxation	n/a	The virtual reality meditative intervention improved patients’ ICU experience with reduced levels of anxiety and depression (estimate = –2.17; 95% CI, –4.23 to –0.106) and depression (estimate = –1.25; 95% CI, –2.37 to –0.129); however, there was no evidence that virtual reality had significant effects on physiologic measures, pain, or sleep
Parke, Hough, and A (2020, United States)* [48]	20 (20/0)	Observational study	3	ICU patients	n/a	The mean intervention time was 29 min	n/a	Xbox Kinect Jintronix software targeting arm, leg, and trunk strength, range of motion, and endurance in 20 adult ICU patients	n/a	VR environment for the delivery of early mobility in patients with critical illnesses was feasible. There were no falls, lines dislodged, or medical events, and this could motivate them to continue. Fatigue was the most common reason for cessation
Faber, Patterson, and Bremer (2013, Netherlands) * [28]	36 (36/0)	Observational study	3	Pediatric and adult burn patients	27.7 (8-57)	One session per day (one and up to seven consecutive days)	wound care session	VR during wound care session (snow World)	n/a	Results from the present study suggest that VR continues to be effective when used for three (or possibly more) treatments during severe burn wound debridement
He et al. (2022, China) [42] *	141 (71/70)	Cohort study	3	ICU patients	52.86 ± 17.96	n/a	ICU stay	5G + VR visitation channels to communicate with the families	No 5G + VR visitation	After 5G + VR visitations, the patients’ HADS scores and the proportion of delirium decreased significantly. No significant difference in the length of ICU stay
Vlake, van Genderen, et al. (2020, Netherlands) [79]	44 (44/0)	Cohort study	3	ICU patients	61 (22-76)	n/a	n/a	Assess the needs, expectations, and wishes of ICU survivors to receive information (brochure, video film/VR)	Patients	Patients suffering from psychological PICS need information, and have no desire to use an information brochure but are willing to receive information via digital content such as a video film/VR (54% of PICS patients)
Naef et al. (2022, Switzerland) * [37]	31 (31/0)	Mixed methods study	3	ICU patients and nursing experts	32 and 73	As long as the participants prefer	ICU stay	Visual and auditory stimulation	n/a	Patients and experts agreed that receiving visual and/or auditory stimuli would benefit patients. Visual stimuli should not exceed 10–15 min, while auditory stimuli should not exceed one hour
Blair et al. (2019, United States) [80]	1	Case report	4	ICU patient with veno-venous extracorporeal membrane oxygenation	21	As long as the patient prefers	Hospital stay	VR-experience with “Pebbles the Penguin”	n/a	The patient reported her anxiety subjectively improved with virtual reality
Esumi et al. (2020, Japan) * [24]	1	Case report	4	ICU patient with compartment syndrome	40	30 min (3 sessions)	Any time	VR program simulates the experience of being at the beach beside a calm sea on a sunny day	n/a	Three sessions of virtual reality analgesic therapy over 2 days produced sustainable analgesic effects, which led to a 25–75% dose reduction in fentanyl administration and the concomitant alleviation of respiratory depression
Chillura et al. (2020, Italy) [74]	1	Case report	4	Post-ICU patient with ICUAW	56	2 exercises, each 55 min (once a day, 6 days a week, for 2 months)	After 2-month conventional training	Physiotherapist-supervised robotic rehabilitation protocol, in addition to the already practiced conventional and respiratory physiotherapy	n/a	At the discharge (6 months after the admission), the patient reached the standing station and was able to ambulate with double support
Vlake et al. (2020, Netherlands) * [52]	1	Case report	4	ICU patient	57	(twice)	17 days after hospital discharge	n/a		One week after receiving ICU-VR, levels of PTSD, anxiety, and depression had normalized, and stayed normalized until 6 months after discharge
Gerber et al. (2017, Switzerland) [72]	37 (37/0)	Proof-of-concept study	n/a	Healthy participants	48 (20-85)	5 min (3 times)	n/a	Three 2D nature videos, every five minutes in length, played as side-by-side videos	n/a	The VR stimulation led to a reduction in heart rate (*p* = 0. 049) and blood pressure (*p* = 0.044). The fixation/saccade ratio (*p* < 0.001) was increased when a visual target was presented superimposed on the videos (reduced search activity), reflecting enhanced visual processing. Overall, the VR stimulation had a relaxing effect as shown in vital markers of physical stress and participants explored less when attending the target
Small et al. (2015, United Kingdom) [81]	25 (25/0)	Randomized controlled trial	n/a	Burn patients	study protocol	Dressing change time	During dressing change	1. Interactive VRET plus conventional analgesics 2. Passive VRET with conventional analgesics	Conventional analgesics alone	Study protocol, the results are not yet available. The study evaluates the effects of VR on pain during and after dressing changes compared with traditional analgesia during dressing changes in a burns unit
Rousseaux et al. (2020, Belgium) * [34]	100 (25/25/25/25)	Randomized controlled trial	n/a	Cardiac surgery patients	study protocol	20 min (twice)	One day before surgery, one day after surgery	3 groups: hypnosis, VR, or virtual reality hypnosis	Daily care only	Study protocol. The study will evaluate the influence of VR applications on patients’ anxiety, fatigue, pain, and phenomenological experience
Naef et al. (2021 Switzerland) [82]	aimed 920	Randomized controlled trial	n/a	ICU patients	study protocol	30 min (3 times a day)	Three times per day, morning, midday, and evening	In addition to the standard ICU care relaxing VR stimulation three times a day	Standard ICU care	Study protocol. The hypothesis is that the VR application may reduce the incidence of delirium
Suvajdzic et al. (2018, United States) * [39]	study protocol	Case–control study	n/a	ICU patients with delirium	study protocol	study protocol	Study protocol	An immersive digital reality-augmenting system consisting of a commercially available VR headset to deliver a calming experience through software-facilitated meditation practice	No VR	Study protocol. The hypothesis is that our virtual reality therapy system would lower the occurrence of delirium in patients admitted to intensive care units
Pediatric intensive care unit										
Hoffman et al. (2019, United States) * [8]	48 (48/0)	Randomized controlled trial	2	Pediatric ICU burn patients	12 (16–17)	12.89 min (4 days)	During wound care: repeatedly alternating between no vr and yes vr every 5 min	VR during the first 5 min of wound care. VR, patients played SnowWorld, an interactive 3D snowy canyon in virtual reality during some portions of wound care	No VR during the first 5 min of wound care	VR significantly reduced children's “worst pain” ratings during burn wound cleaning procedures in the ICU on Day 1. Worst pain during No VR = 8.52 (SD = 1.75) vs. during Yes VR = 5.10 (SD = 3.27), *t*_(47)_ = 7.11, *p* < 0.001, SD = 3.33, CI = 2.45–4.38, Cohen's *d* = 1.03 (indicating large effect size). Patients continued to report the predicted pattern of lower pain and more fun during VR, during multiple sessions
Agasthya et al. (2020, United States) * [19]	15 (7/8)	Randomized controlled trial	2	Pediatric residents	n/a	19 min	Training	9-min VR tutorial that outlined the steps involved in preparation for pediatric airway intubation	The non-VR group listed intubation steps from memory	The VR group had seven trainees (47%) and scored similarly to the other group based on checklist items (50.5% vs 50.8%, *P* = 1)
Umoren et al. (2021, United States) [83]	274 (91/95/88)	Randomized controlled trial	2	Health care providers	38 ± 9	n/a	Training	VR simulation + digital guide (*n* = 91)	Video + digital guide (*n* = 95) Digital guide only (*n* = 88)	Neonatal resuscitation skills pass rates were similar among the groups at the 6-month follow-up bag and -mask ventilation (BMV) skills check (VR 28%, video 25%, control 22%, *p* = 0.71), objective structured clinical examination (OSCE) A (VR 76%, video 76%, control 72%, *p* = 0.78) and OSCE B (VR 62%, video 60%, control 49%, *p* = 0.18). Relative to the immediate postcourse assessments, there was greater retention of BMV skills at 6 months in the VR group (− 15% VR, *p* = 0.10; − 21% video, *p* < 0.01, –27% control, *p* = 0.001). OSCE B pass rates in the VR group was numerically higher at 3 months (+ 4%, *p* = 0.64) and 6 months (+ 3%, *p *= 0.74) and lower in the video (− 21% at 3 months, p < 0.001; − 14% at 6 months, p = 0.066) and control groups (− 7% at 3 months, *p* = 0.43; − 14% at 6 months, *p* = 0.10). In a follow-up survey, 95% (*n* = 65) of respondents in the VR group and 98% (*n* = 82) in the video, group would use their assigned intervention again
Yang et al. (2022, South Korea) * [17]	83 (29/28/26)	Non-randomized trial	3	ICU staff	n/a	50 min	Training	Neonatal resuscitation gamification program using immersive virtual reality	Only resuscitation program lectures	Neonatal resuscitation knowledge [F (2) = 3.83, *p* = 0.004] and learning motivation [F (2) = 1.79, *p *= 0.025] were significantly higher in the virtual reality and simulation groups than in the control group. Problem-solving ability [F (2) = 2.07, *p* = 0.038] and self-confidence [F (2) = 6.53, *p* < 0.001] were significantly higher in the VR group than in the simulation and control groups. Anxiety [F (2) = 16.14, *p* < 0.001] was significantly lower in the simulation group than in the VR and control groups
Badke et al. (2019, United States) * [54]	28 (28/0)	Observational study	3	PICU patients	9 (7–13.3)	max. 15 min	n/a	Choice of developmentally appropriate VR experiences, ranging from serene natural landscapes, such as safari or scuba diving, to more thrilling videos, such as snowboarding and roller coasters	n/a	One hundred percent of participants enjoyed using virtual reality, and 84% reported a preference to use virtual reality for a longer duration. One hundred percent of parents agreed that their child enjoyed using virtual reality and 100% enjoyed watching their child use virtual reality
Tallent et al. (2021, United States) * [59]	9 (9/0)	Observational study	3	Caregiver of pediatric ICU patients	31.1 [23–40]	max. 15 min	On medical rounds	Join medical rounds of their kids in PCICU	n/a	VR can be successfully implemented for family engagement without increased burden on staff. It did not increase rounding time (*p* = 0,673), and workload impact perceptions improved after intervention (*p* = < 0.001)
Farra et al. (2019, United States) * [18]	93 (n/a)	Observational study	3	Staff related to neonatal ICU evacuation	25–31	10 min (at 0, 4, 8, and 12 months)	4 times: 0, 4, 8, and 12 months	Virtual reality simulation (VRS) emergency evacuation training	web-based clinical updates (CU)	The VRS and CU groups did not statistically differ based on the scores on the Cognitive Assessment or perceived self-efficacy. The virtual reality group’s performance in the live exercise was statistically (*P* < 0.0001) and clinically (effect size of 1.71) better than that of the CU group
Ralston et al. (2021, United States) * [15]	6 (6/0)	Observational study	3	Pediatric cardiac critical care physicians	n/a	6 min (range 4–8 min)	n/a	Pilot test of VR’s feasibility for educational and practice improvement efforts	n/a	VR might be more effective in trail training specifications Clinicians previously unfamiliar with VR can engage in simulated acute clinical scenarios common to the pediatric CICU
Abdulsatar et al. (2013, Canada) * [57]	8 (8/0)	Observational study	3	Pediatric ICU patients	11 (3-18)	54.5 (15, 224) min	2 times over the 2-day intervention period in PICU	Wii™ play	n/a	VR can be safely used with critically ill children Upper limb activity during Wii™ was significantly greater than the average daily activity (*p* = 0.049). Grip strength did not change significantly from baseline (*p* = 0.20)
Yu et al. (2021, Republic of Korea) * [16]	50 (25/25)	Observational study	3	Nursing students	22.40 ± 1.05	40 min	Training	Virtual reality simulation of three scenarios: basic care, feeding management and skin care and environmental management for prevention of neonatal infection	routine neonatal intensive care unit practice	Compared to the control group, the experimental group showed significantly greater improvements in high-risk neonatal infection control performance self-efficacy (t = -2.16, *p* = 0.018) and learner satisfaction (t = -5.59, *p* < 0.001)
Badke et al. (2022, Chicago) * [55]	115 (115/0)	Cross-sectional study	3	Critically ill children	10 (6–13)	10 min (7-17)	ICU stay	VR headset was used to deliver 360-degree immersive experiences	n/a	83% of participants smiled, 36% laughed and 79% were highly engaged while using VR. 92% of parents reported that VR calmed their children, and 78% of participants felt that VR was calming. HRVi Minimum scores were significantly higher during VR
Lai et al. (2021, United States) * [58]	2	Case report	4	PICU patients	Case 1:15 Case 2: 13	Case 1: 18 min (SD 11) per session, 4 sessions Case 2: 35 min (SD 7) per session, 2 sessions	Between usual care, when tolerable and requested by the participant	VR gaming sessions with active games (e.g., boxing, rhythmic movement to music, and exploratory adventure) and nonactive games (e.g., racing and narrative adventure)	n/a	The findings of this study suggest that VR gaming with HMDs and adaptive software is likely a feasible supplement to usual care for adolescents within the PICU, and these findings warrant further investigation. Recommendations for future studies aimed at incorporating VR gaming during early mobilization are presented herein
Hemphill et al. (2021, United States) [73]	1	Case report	4	Pediatric ICU patient	n/a	n/a	During physical therapy sessions	VR to encourage the child to engage in physical therapy sessions	n/a	Virtual reality encouraged the child to engage in physical therapy sessions, participate for greater durations, and directly address barriers to discharge
Scapin et al. (2017, Brazil) [84]	2	Case report	4	Pediatric burn patients	8, 9	15 min, 35 min, 25 min	Dressing change during balneotherapy	The child used VR to watch a game that simulated a roller coaster	n/a	The use of goggles was easy to apply and well-accepted by the children, and had a relevant effect reducing pain (pain face: case 1: 10/10 during a dressing change, 4 during VR use vs. case 2: 4 during dressing; 0 during use VR,)
Kucher et al. (2020, United States) * [56]	6 (6/0)	Proof-of-concept study	n/a	Total pancreatectomy and islet auto-transplant (TPIAT) surgery patients	8–18	25.6 min ( 9–90 min), individual preference (between 1 – 5 times)	After surgery	After surgery nature-based theme VR	n/a	Initial quantitative scoring systems suggest overall improvement in symptom management, and reactions by both patients and their parents were overall positive

**Table 2:** Systematic review of augmented reality in intensive care medicine

Authors (year, country)	Sample size (intervention/ control)	Study type	Quality of evidence [75]	AR user	Age (mean ± SD) median (range)	Aim	The timing of applying AR	Experimental group intervention	Control group intervention	Result
Dias et al (2021, USA) * [68]	15/15/15	Randomized controlled study	2	ICU nurses (neonatal)	n/a	Endotracheal intubation of a puppet	Training	AR-assisted video laryngoscopy (AVL) with a magnified video of the airway into the intubator’s visual field	Direct laryngoscopy (DL) or indirect video laryngoscopy (IVL)	The DL group successfully intubated on 32% of attempts compared to 72% in the IVL group and 71% in the ARVL group (*P* < 0.001). The DL group intubated the esophagus on 27% of attempts, whereas there were no esophageal intubations in either the IVL or ARVL groups (*P* < 0.001). The median (interquartile range) time to intubate in the DL group was 35.6 (22.9–58.0) seconds, compared to 21.6 (13.9–31.9) seconds in the IVL group and 20.7 (13.2–36.5) seconds in the ARVL group (*P* < 0.001)
Huang et al. (2018, USA) * [60]	16/16	Randomized controlled study	2	ICU clinicians and trainees	29.8 ± 7.8	Central venous catheters in the manikin	Training	The AR simulation group had a 5–10-min hands-on instructional course to allow familiarity with the AR equipment. During central line placement, a video was displayed repeating essential steps	Using the ultrasound to attempt an internal jugular vein central line insertion on a manikin	No difference regarding the meantime for placement or procedure time, but a significantly higher adherence level between the two groups favoring the AR group (*p* = 0.003)
Heo et al. (2022, Republic of Korea) * [64]	15/15	Randomized controlled study	2	Nurses	24—53	AR-based self-learning A platform for novices to set up a ventilator without on-site assistance	Training	The AR group was guided by AR-based instructions and requested assistance with the head-mounted display	The manual group used a printed manual and made a phone call for assistance	Fewer participants requested assistance in the AR group compared to the manual group and the number of steps that required assistance was lower in the AR group. A higher rating in predeveloped questions for confidence and suitability of the method
Alismail et al. (2019, USA) * [63]	15/17	Controlled study	3	ICU clinicians and trainees	30 ± 7.8	Endotracheal intubation of a puppet	Training	Intubation of a puppet with AR glasses head mount display that displayed the essential steps	Intubation (of a puppet)	The AR group took longer median (min, max) time (seconds) to ventilate than the non-AR group (280 (130,740) vs 205 (100,390); η 2 = 1.0, *p* = 0.005, respectively). Similarly, there was a higher percent adherence to the NEJM intubation checklist (100% in the AR group vs 82.4% in the non-AR group; η2 = 1.8, *p* < 0.001)
Fumagalli et al. (2017, Italy) * [61]	56/47	Controlled study	3	ICU personnel	74 ± 12	AR-assisted venous puncture using near-infrared electromagnetic radiation in elderly ICU patients	Admission to ICU	Venous puncture with AR	Standard venous puncture	The use of the novel NIR-based device is safer and more psychologically tolerable (*p* = 0,038), and it is not associated with an increase in procedure length (standard: 7.0 ± 3.9 vs. AR: 8.0 ± 5.8 min, *p* = 0.173) or several attempts (standard: 1.3 ± 0.6 vs. NIR-BD: 1.2 ± 0.6, *p* = 0.361). Hematoma development after venipuncture was directly associated with a significant reduction in AR group patients (OR 0.21, 95% CI 0.05–0.80, *p* = 0.022)
Bloom et al. (2022, USA) [85]	30/0	Observational study	3	Pediatric cardiologists or intensivists	n/a	Venous puncture	Training	Venous puncture with mixed reality	Conventional US	Reduction in the number of needles repositions (*P* = 0.03), improvement in quality of access as measured by distance (*P* < 0.0001) and angle of elevation (*P* = 0.006), faster time to access (*P* = 0.04), fewer number of both access attempts (*P* = 0.02) and a number of needles repositions (*P* < 0.0001) compared to conventional US. Postparticipant surveys showed high levels of usability (87%) and a belief that MantUS may decrease adverse outcomes (73%) and failed access attempts (83%)
Zackoff et al. (2021, USA) * [66]	84/0	Observational study	3	ICU clinicians and trainees	n/a	Assessing a decompensating patient in a training situation	n/a	All teams completed two pieces of training: (1) traditional training using a manikin and (2) AR-enhanced training using a manikin plus an AR patient	n/a	AR improved the ability to assess the patient's mental status, respiratory status, and perfusion status (all *P* < 0.0001) during AR in comparison to TT. Similar findings were noted for the recognition of hypoxemia, shock, apnea, and decompensation (all *P* ≤ 0.0003) but not for the recognition of cardiac arrest (*P* = 0.06)
Yamada et al. (2019, Japan) * [67]	n/a	Observational study	3	Perfusionist	n/a	AR experiences using the back camera of a smartphone or tablet. We can also build our instrument with custom visualization and data analysis	n/a	AR program for Extracorporeal circulation technology	n/a	Results indicate that future perfusionists may study AR in classrooms because there is an intimate relationship between virtual and physical objects. This AR technology for ECC is cost-effective and relatively easy to construct
Scquizzato (2020, Italy) * [69]	n/a	Observational study	3	ICU or emergency personnel	n/a	A smartphone application with augmented reality for estimating weight in critically ill pediatric patients	n/a	A smartphone app that estimates child weight using the smartphone camera and augmented reality (AR) by implementing a virtual 3D tape	n/a	This app could improve a child’s weight estimation by implementing and training a machine learning regression model that features measurement data from the app, child gender, and habitus
Morillas Perez et al. (2023, Spain) * [62]	6/0	Cohort study	3	ICU personnel	n/a	AR-assisted vascular puncture	Training	Simulation of an AR-assisted vascular puncture on an experimental model	n/a	37 with 33 punctures were successful and after technical improvements, 39 with 38. There are no significant differences between the operators and between the ultrasound scanners. AR-based punctures provide greater accuracy, and greater comfort by freeing the hands and keeping the gaze on the working field
Gan et al (2019, USA) * [65]	6	Case series	4	ICU personnel	n/a	AR-assisted percutaneous dilatational tracheostomy	Tracheostomy	Augmented reality during percutaneous dilatational tracheostomy placement	n/a	“Good success and excellent user feedback”

*Study cited in the results section

## Part 1: VR

### VR as a tool for health care providers to improve clinical practice

#### Adult intensive care medicine

**Fig. 2:**
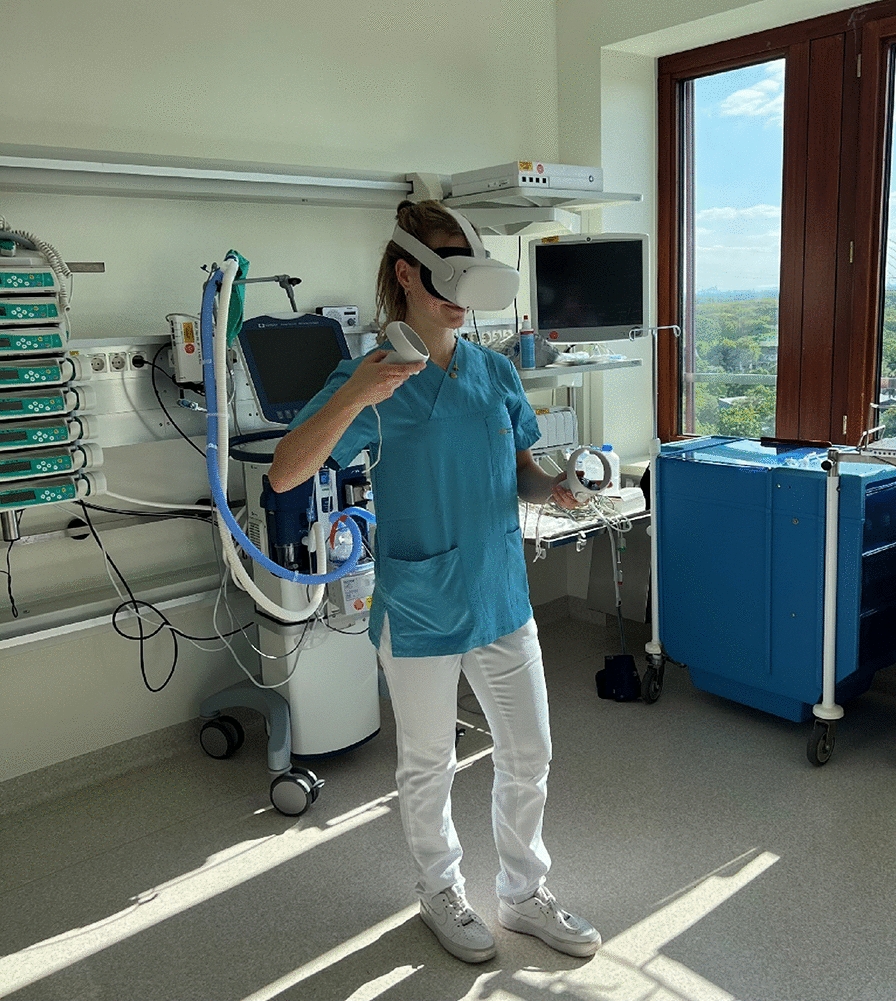
VR application for the training of health care providers

VR might assist in educating and training healthcare professionals [2] (Fig. 2) as intensive care treatment strategies are often complex and require not only theoretical knowledge but also practical preparation. In a randomized controlled trial of 381 participants, Nas et al. evaluated the value of VR for learning cardiopulmonary resuscitation (CPR). They reported comparable chest compression rates but an inferior compression depth compared with face-to-face training [11]. The research on VR/AR in this field is generally very heterogeneous [12]. Wolff et al. developed a VR training environment to improve the traditional training for extracorporeal membrane oxygenation (ECMO) [5]. Bronchoscopy is another important tool for diagnostic and therapeutic purposes in ICU patients and performing this procedure can be challenging. Colt et al. created a virtual reality bronchoscopy simulation. Through the acquired skills after VR training, five novice physicians were comparable to four experienced physicians regarding dexterity, speed, and accuracy in the model [13]. In a prospective randomized study with 60 healthcare providers, Chiang et al. evaluated 15-min VR-based learning on tracheostomy care. The use of VR materials increased significantly participants' self-efficacy (increased familiarity, more self-confidence, and less anxiety) and the positive impact persisted until 3 to 4 weeks later [14].

### Pediatric intensive care unit

Caring for patients in the neonatal and pediatric intensive care units (PICU) can be particularly challenging [15], and aspects such as performance, knowledge, self-efficacy, and learner satisfaction are of great importance. In terms of these endpoints, Yu et al. evaluated the effects of a VR simulation program on nursing students. In three scenarios, the interventional group (*n* = 25) experienced a 40-min VR simulation and routine practice, and the control group (*n* = 25) only did routine practice. The use of VR resulted in a significant increase in high-risk neonatal infection control performance, self-efficacy, and learner satisfaction [16]. Yang et al. investigated in a non-randomized controlled study the impact of VR neonatal resuscitation program among others on knowledge, problem-solving, or degree of anxiety [17]. The VR group (*n* = 29) participated in a neonatal resuscitation gamification program, while the simulation group (*n* = 28) participated in high-fidelity simulations of neonatal resuscitation and online lectures. The control group (*n* = 26) had only online lectures on neonatal resuscitation. VR and simulation groups achieved significantly higher levels of neonatal resuscitation knowledge and learning motivation than the control group. Furthermore, VR application was found to be effective in increasing problem-solving ability and self-confidence compared to the others groups. However, anxiety was lowest in the simulation group. Ralston et al. investigated VR-based simulation of two scenarios: ectopic junctional tachycardia and low cardiac output syndrome in the early postoperative period and acute respiratory failure in a patient with suspected coronavirus disease [15]. All six pediatric cardiac critical care physicians successfully navigated the VR environment and met the critical endpoints such as connect the patient to the pacemaker and correctly overdrive pace or intubate the patient and connect to the ventilator. Farra et al. compared the success of VR training versus web-based clinical updates for emergency evacuation in a newborn ICU. Although there was no significant difference in terms of cognitive assessments and self-efficacy, the VR group performed statistically and clinically better in the live exercise [18]. Agasthya et al. evaluated a VR tutorial for endotracheal intubation. Participants of the interventional group completed a 19-min immersive guiding and the control group listed the steps from memory. Both groups demonstrated their skills with traditional manikins and were scored on a 24-point checklist. There was no significant difference between the groups [19].

### VR as a tool for healthcare providers to reduce stress

Stress is a common phenomenon in the intensive care unit for both patients and health care providers. In a study with 66 ICU nurses investigated Nijland et al. the effect of VR on perceived stress levels. Sixty-two percent of the ICU nurses, who used VR-Relaxation during their breaktime reported VR to be helpful to reduce stress [20]. Bodet-Contentin et al. also showed in a study of 88 intensive unit caregivers that the use of VR could improve the efficiency of the breaks [21].

### Patient experiences with VR during and after ICU-stay

#### Adult intensive care

**Fig. 3:**
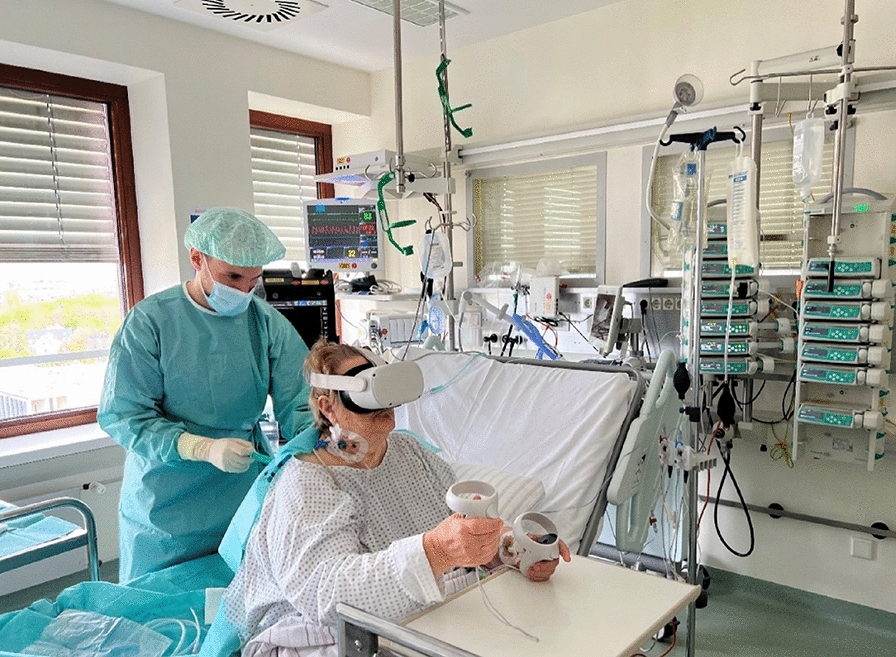
VR application during ICU treatment to distract the patients

From the patients' perspective, intensive care treatment is associated with a number several symptoms such as pain [1]. If one now modulates attention, environmental conditions, and mood with VR, this can reduce the attention devoted to pain [22] (Fig. 3). Mosso-Vázquez et al. used VR to present different immersive environments such as Cliff or Dream Castle to 67 patients after cardiac surgery [23]. The results were evaluated with a Likert scale and almost 90% of the patients reported a decreased level of pain experienced post-therapy with VR. Esumi et al. evaluated VR in a patient whose pain after a fasciotomy for acute compartment syndrome could not be adequately controlled and opioid-related side effects, such as respiratory depression, have occurred. The use of VR led to a 25–75% dose reduction in fentanyl administration and the concomitant alleviation of respiratory depression [24]. In a randomized, prospective study of 200 cardiac surgery patients, Laghlam et al. demonstrated that VR application was equivalent to conventional treatment with oxygen and nitrous oxide in terms of reported pain scores during removal of chest tube [25]. Markus et al. focused on the technical and procedural feasibility of VR in daily routine and showed that the VR application takes almost an hour for setup, instruction, VR therapy, and cleaning. Especially in smaller centers such programs would be difficult to implement due to lack of staff and resources [26]. Hoffman et al. demonstrated in their study with 11 burn-injured patients the positive effects of 3-min VR application during wound care on pain relief and a positive correlation between the immersive strength of VR and its pain-relieving effect [27]. However, Faber et al. showed that the effect would be less after three consecutive days [28].

Due to discomforts such as aggressive noises, lights, and a lack of information, the intensive care units are often associated with negative feelings such as anxiety or stress for patients [7]. Merliot‑Gailhoustet et al. investigated in a randomized trial E‑CHOISIR (Electronic‑CHOIce of a System for Intensive care Relaxation) the effects of different electronic relaxation devices on the reduction of overall discomfort, pain, anxiety, dyspnea, thirst, lack of rest feeling and stress in sixty ICU-patients. The patients received four relaxation sessions (standard relaxation with TV or radio, music therapy, and two VR systems with real or synthetic motion pictures). In the group with synthetic motion pictures the overall discomfort, pain, and stress could be significant decrease, while the real motion pictures were associated with a reduction in lack of rest. Both VR-Systems led to a significant decrease in anxiety. Three adverse events might occur: claustrophobia, dyspnea, and agitation. However, in general cybersickness (occurrence of symptoms such as headaches or nausea during VR use) rarely occurred [29].

Haley et al. evaluated in a pilot study 5-min VR sessions in 10 mechanically ventilated patients. VR therapy proved to be a potential means of managing anxiety in this patient group without the occurrence of predefined safety events or cybersickness [30]. The quality of sleep could also be positively influenced by the use of VR. In a study with 100 patients, it was shown that due to the VR application, the sleep quality was significantly better but the total sleep time and light sleep time did not differ between the groups [31].

Hypnosis has been used in the management of acute and chronic pain for a long time [32]. Rousseaux et al. tested a “virtual reality hypnosis” in patients undergoing cardiac surgery comparing VR to control patients, hypnosis without VR, and VR without hypnosis. All four techniques were used one day before and one day after surgery [33, 34]. However, in their randomized-controlled study with 100 patients, there were no significant differences regarding the outcome measures (anxiety, pain, fatigue, relaxation, physiological parameters, and opioid use) between the groups [35].

To evaluate the usefulness of VR for reducing sensory overload and deprivation in the ICU Jawed et al. put VR goggles on 15 ICU patients for 15 min and exposed them to relaxing beach videos with nature sound effects. Most patients tolerated the headsets well and reported the positive effects of VR therapy on anxiety and stress [36]. Naef et al. investigated how long visual and auditory stimuli should be provided to intensive care unit patients. In their study, visual stimuli should not exceed 10 – 15 min, while auditory stimuli should not exceed one hour to prevent negative side effects [37].

Suvajdzic et al. used a combined approach to prevent delirium in ICU patients—The DREAMS system (Digital Rehabilitation Environment-Altering Medical System) [38], which combined an immersive digital reality acquisition system with a measurement system. The VR environment consisted of a commercially available VR headset. The measurement was sophisticated: it includes physiologic sensors 3-axis wearable accelerometers, a video camera, and environmental sensors for light and noise exposures for measurement of movement, physiologic and emotional responses to assess the movement, physiologic and emotional responses. In addition, an electroencephalogram sensor measures the sleep quality and response to therapy [39]. The DREAMS system has so far only been used in a small feasibility study with 59 non-intubated ICU patients and was well-received but there was no significant effect on physiologic measures, pain, or sleep [40].

Family support also plays a big role for the patients in the ICU [41]. Therefore, He et al. used the fifth generation plus virtual reality (5G + VR) equipment to establish visitation channels for patients and their families during the COVID-19 pandemic. They showed in a cohort study with 141 ICU patients that after 5G + VR visitations, the Hospital Anxiety Depression Scale (HADS) decreased significantly, along with a significant reduction in the proportion of delirium [42].

ICU patients often experience not only delirium but also other neurocognitive impairments [43]. In this context, Turon et al. examined in a pilot study the benefits of VR-assisted early neurocognitive stimulation in 20 critically ill adult patients [44]. The simulation includes a virtual avatar that accompanies patients, helped them orient to time, delivered instructions, motivated them to complete exercises, and encouraged them to relax. This application was found to be feasible, safe, and reliable, and stimulated cognitive functions. Navarra-Ventura et al. evaluated also a VR-based neurocognitive intervention during ICU stay in 34 critically ill patients. A 1-month follow-up that these patients had better working memory scores and showed up to 50% less non-specific anxiety and depression compared to the control group [45].

Early mobilization of ICU patients improves patient outcomes and reduced hospital stay length [46]. Gomes et al. used Nintendo Wii™ in 60 adult ICU patients to increase their physical activity [47]. Activity levels were light to moderate on a modified Borg scale and a majority of patients expressed a desire to play the videogame during their upcoming physical therapy sessions. The study from Parke et al. utilized a similar approach: Xbox Kinect Jintronix software targeting arm, leg, and trunk strength, range of motion, and endurance in 20 adult ICU patients [48]. Most patients found the activity enjoyable, and easy to understand, as well as motivating to continue participating.

ICU stay constitutes a considerable psychological burden for patients. In several studies, Valke et al. investigated the effects of ICU-specific virtual reality on mental health [49–52]. In one of them with 104 patients the group evaluated three and six months after ICU treatment, repetition of 14-min VR modules about ICU treatment improves subjective well-being and quality of life. VR resulted in a reduction of post-traumatic stress disorder, and depression scores, and the effect was still present six months after exposure. Although the mental health was also initially better this effect was no longer observed after six months.

### Pediatric intensive care unit

The stay in the pediatric intensive care unit (PICU) can be an emotional and stressful experience for both children and parents [53]. In a pilot study with 32 critically ill children, Badke et al. investigated the feasibility and satisfaction of virtual reality in the PICU. All participants enjoyed using the technology, and 84% expressed interest in using it for a longer period. The positive effects were also observed among the parents, with 100% reporting satisfaction while watching their children use virtual reality. Moreover, parents reported that their children were calmed by VR [54]. In another study by this group with 115 critically ill children, the positive influence of VR on engagement and physiologic effects such as heart rate variability was confirmed [55].

Kucher et al. [56] and Hoffmann et al. [8] evaluated VR for better pain management and both were able to show positive effects. Abdulsatar et al. investigated the feasibility and safety of using Nintendo Wii™ in a pilot-trials with 12 critically ill children [57]. The application improved upper limb activity but grip strength did not change significantly from baseline. Lai et al. used VR on two adolescents suffering from Covid-19. The patients could choose from various active games such as boxing and non-active games such as racing. The authors conclude that VR gaming improved participants’ affect and alertness, motivating them to engage more in early mobilization therapy [58].

The hospital-induced separation between the child and the family is difficult for both sides. Therefore, Tallent et al. also established a VR-based virtual visit and the staff surveys showed that the application did not lead to an increased duration of the visit. Endpoints on parental perception are not reported, but VR appeared to be very well accepted by the treatment team in this study [59].

## Part 2: AR

### AR as a tool to assist ICU procedures

#### Adult intensive care unit

AR can also help health care providers in the implementation of procedures in the ICU. Huang et al. evaluated the AR application during central venous line placement. The AR intervention consisted of a 5- to 10-min hands-on instructional course to allow familiarity with the AR equipment and—during central line placement in a manikin—a video that repeated essential steps. There was no difference between the groups regarding the meantime for placement or procedure time, but a significantly higher adherence level to the checklist between the two groups favoring the AR group was observed [60]. Fumagalli et al. evaluated the value of AR for venous puncture in 103 ICU patients. The use of AR reduced the incidence of hematomas and anxiety levels but did not reduce the duration of the procedure or the number of attempts [61]. Morillas Perez et al. also confirmed the positive influence of AR on vascular puncture in a study with 6 operators, who performed a total of 76 punctures. AR application resulted in higher accuracy and better quality of the images and eliminated variability between operators and sonographers. Furthermore, it provided more comfort as the hands are free and the view remains focused on the work area [62]. In a controlled trial with 32 ICU trainees, Alismail et al. investigated the use of AR during the endotracheal intubation of a manikin. The use of AR, where the essential steps were repeated, resulted in a longer need of time to intubate and ventilate but demonstrated higher compliance with the checklist [63]. Heo et al. randomized 30 nurses without experience in mechanical ventilation into 2 training groups: with or without AR. Compared to the control group, the AR group requested less assistance and showed higher self-confidence [64]. Gan et al. evaluated in 6 cases the AR for percutaneous dilatational tracheostomy and again it was confirmed that this new technology allowed the procedure to be carried out successfully [65]. A pilot study by Zackoff et al. evaluated AR in two critical situations. AR not only improved the ability to assess many factors such as the mental or respiratory status of the patient, but also had a positive impact on the recognition of critical situations such as shock, apnea, and hypoxemia. However, the detection of cardiac arrest was not significantly better [66]. To improve the training of future perfusionists in the field of extracorporeal circulation (ECC) Yamada et al. developed an AR program for smartphones or tablets [67]. The AR training might be beneficial for future perfusionists, but currently there has not yet been a clinical study examining the use of the app.

### Pediatric intensive

#### Care unit

Dias et al. also evaluated AR to improve performing endotracheal intubation. Forty-five participants were randomly divided into three groups and used for intubation on a manikin either direct laryngoscopy or indirect video laryngoscopy or AR-assisted video laryngoscopy. AR-assisted video laryngoscopy was comparable to indirect video laryngoscopy but resulted in increased safety compared with direct laryngoscopy [68]. The dosage of the drugs used during critical situations in the ICU is often based on weight. Therefore, Scquizzato et al. developed a smartphone app that estimates child weight using the smartphone camera and augmented reality (AR). So far, it has not been evaluated in clinical trials [69].

## Limitations

Although the number of studies about the use of VR or AR significantly increases year by year, attempts at systematic synthesis of evidence such as the present study are limited by scarcely comparable methods, devices, and protocols [70]. A limited number of prospective randomized controlled trials are currently available in this field and the data are generally very heterogeneous. Thus, quantitative synthesis by meta-analysis and the use of methods to assess the risk of bias in the included studies is hardly possible. Several sources of bias could affect the validity and reliability of studies investigating the use of VR and AR in the ICU. The sample size of the studies is often small and not representative of the overall population of ICU patients, what increases the selection bias. Moreover, the inclusion of older adults may be limited by the fact that they are less familiar with new technologies such as VR/AR and may be hesitant or resistant to trying these innovation methods. It is important that VR/AR interfaces can accommodate age-related changes, such as visual impairments, hearing impairments, and decreased dexterity, to facilitate use the technologies. The performance bias can be high because most studies are not blinded and this can influence the behavior of participants. Furthermore, the outcomes are in most studies subjective and dependent on observation. Establishment of objective evaluation criteria is necessary to improve these aspects. However, there are some subjects such as post-traumatic disorders that are inherently complex and multifaceted, making it difficult to develop such criteria that capture all relevant factors. In addition, a common challenge is the diversity of applied VR and AR systems. This can affect not only the complexity of the application but also the tolerance of the users. VR/AR tools can provide varying levels of user comfort, performance, and immersion. This can make it difficult to reproduce the data, which can reduce the reliability and comparability of the research results. Furthermore, only a few studies describe exactly how time-intensive the application of the technologies can be. This, combined with differences in cost and accessibility, may impact the widespread application of VR/AR.

The present systematic review found the same difficulties for intensive and critical care medicine. In upcoming studies, the protocols should be harmonized as far as possible to expand significant clinical knowledge. The development of a core outcome set plays an important role for future systematic research about VR and AR. This would allow better comparability of studies, improve the quality and relevance of results, and facilitate evidence synthesis and meta-analyses. By providing this information, it would be even more possible to generalize the results and to understand the benefits and limitations of VR in the clinical setting.

## Summary

This review showed that VR and AR offer new possibilities for many aspects of daily intensive care medicine. There are several approaches to supporting traditional clinical training and taking medical education to the next level. They provide a safe environment to practice procedures such as bronchoscopy [13], without risking harm to patients. In the ICU, the health care providers often have to make quick decisions, and by simulating different scenarios using VR such as low cardiac output syndrome [15], the critical decision-making skills can be improved. In addition, the technologies can positively influence aspects such as knowledge or self-efficacy [14, 16], and the stress level of hospital staff [20]. However, the studies also have shown that VR/AR applications do not lead to a significant improvement in the performance of invasive procedures such as central line placement [60]. Furthermore, VR-based CPR training compared with traditional training provided inferior results [71].

Although there is currently limited data on clinically relevant outcomes, combining traditional training with VR/AR applications may be the way to achieve the best results in daily clinical practice.

Several studies confirmed that VR also might be an effective tool for pain management. VR allows us to generate a virtual environment to distract patients from their pain for example after surgery [23] or during wound care [8, 27]. As a result, VR therapy can reduce the need for pain medication and thus prevent the occurrence of undesirable side effects of traditional therapy [24]. Furthermore, the use of VR can lower the stress level and reduce anxiety during the stay in the ICU [36, 72]. This can have a positive impact on sleep quality [31], development of delirium [42], and cognitive impairment [45]. Six studies have also shown that VR can help during rehabilitation [47, 48, 57, 58, 73, 74]. The application led to an increase in activity and was well tolerated by the patient without the occurrence of adverse events such as falls.

It is necessary to pay attention to the duration of the application of these technologies because overstimulation can negatively affect the outcome of patients [37]. Furthermore, cybersickness may occur during the application [29]. Lastly, the implementation of these technologies into clinical practice requires a significant investment of time by ICU staff [26], which also may reduce readiness to use them.

Overall, while VR is not a substitute for established therapy, it can be a useful tool in combination with other treatments to improve the patient's stay in an intensive care unit.

## Conclusion

Augmented reality (AR) and virtual reality (VR) are no longer the domains of the science fiction world. We are on the verge of making virtual and augmented reality mainstream in the field of medicine and critical care has the potential to be at the forefront of this evolution. However, we cannot forget that VR and AR are not intended to distract us from the patient. They are provided to complement and optimize, but not replace the relationship between a health care provider and a patient. Furthermore, these are still in the research and development phase. Our involvement in this process is important to ensure that these technological developments are made in the best interest of our patients. This makes it possible to provide the best care and to improve the quality of the hospital stay in the ICU.

## Data Availability

The anonymized data can be requested from the authors if required.

## See Table 3

**Table 3:** Search strategy

**Search terms** *(pubmed.ncbi.nlm.nih.gov)*	**Results** ^**1**^	**Included** ^**2**^
“VR” AND “ICU”	56	20
“virtual reality” AND “ICU”	73	26
"virtual reality" AND "critical care"	142	34
“virtual reality” AND “intensive care unit”	61	24
“AR” AND “ICU”	383	3
"augmented reality" AND "ICU"	1	1
"augmented reality" AND "critical care"	21	5
"augmented reality" AND "intensive care"	24	5
“mixed reality” AND “ICU”	13	8
“mixed reality” AND “critical care”	8	2
“mixed reality” AND “intensive care”	4	0

^1^Some publications appeared for multiple search terms. Therefore, the number of included studies is higher than the number of studies listed in the table

^2^Conducted until 1st March 2023

## Abbreviations

3D: Three dimensional
5G: Fifth generation
AR: Augmented reality
CPR: Cardiopulmonary resuscitation
ECC: Extracorporeal circulation
ECMO: Extracorporeal membrane oxygenation
HADS: Hospital Anxiety Depression Scale
HMD: Head-mounted display
ICU: Intensive care unit
IPD: Interpupillary distance
PICU: Pediatric intensive care unit
PICS-F: Post-intensive care syndrome-family
PRISMA: Preferred Reporting Items for Systematic Reviews and Meta-Analyses
TAVR: Transcatheter aortic valve replacement
VR: Virtual reality
